# Cost-effectiveness of fuzuloparib compared to routine surveillance, niraparib and olaparib for maintenance treatment of patients with germline BRCA1/2 mutation and platinum-sensitive recurrent ovarian carcinoma in China

**DOI:** 10.3389/fphar.2022.987337

**Published:** 2023-01-04

**Authors:** Jing Nie, Huina Wu, Lei Sun, Yanjiao Ding, Yepeng Luan, Jiyong Wu

**Affiliations:** ^1^ Department of Pharmacy, Shandong Second Provincial General Hospital, Jinan, Shandong, China; ^2^ Department of Medicinal Chemistry, School of Pharmacy, Qingdao University Medical College, Qingdao University, Qingdao, Shandong, China

**Keywords:** fuzuloparib, niraparib, olaparib, cost-effectiveness, maintenance treatment, ovarian cancer, BRCA1/2 mutation

## Abstract

**Background:** Maintenance therapy with the poly (ADP-ribose) polymerase inhibitors (PARPis) for platinum-sensitive recurrent ovarian carcinoma (OC) have proven to be effective compared with placebo. We aimed to evaluate the cost-effectiveness (CE) of maintenance fuzuloparib compared to routine surveillance (RS), niraparib and olaparib for platinum-sensitive recurrent OC from the Chinese healthcare systems.

**Method:** A partitioned survival model with three-state (progression-free, progressed, death) was constructed utilizing TreeAge Pro 2011 software to evaluate the economic value of fuzuloparib, niraparib and olaparib maintenance treatment for platinum-sensitive recurrent OC based on the clinical data derived from FZOCUS-2, ENGOT-OV16/NOVA and ENGOT-Ov21/SOLO2. Transition probabilities were estimated from the reported survival probabilities in those trials. Cost and health preference data were derived from the literature. The quality-adjusted life-years (QALYs) and lifetime costs were measured for this analysis. A 5 years horizon and 5%/year discount rates were used. One-way analysis, and probabilistic sensitivity analysis (PSA) were performed to explore the model uncertainties.

**Results:** Total cost of fuzuloparib, niraparib and olaparib were $31628.10, $48183.48 and $54605.54, whereas they had an incremental cost-utility ratio of $31992.69, $32216.08 and $23359.26 per additional progression-free survival (PFS) QALYs gained compared with RS, relatively. Model showed that maintenance fuzuloparib achieved at least an 85.5% probability of CE at the threshold of $37654.50/QALY. One-way sensitivity analysis revealed that the results were sensitive to the PFS and the price of medicines.

**Conclusion:** Fuzuloparib was less cost-effective for patients with germline BRCA1/2 mutation and platinum-sensitive recurrent OC compared to olaparib, but was superior to niraparib from the Chinese healthcare systems perspective.

## Graphical Abstract

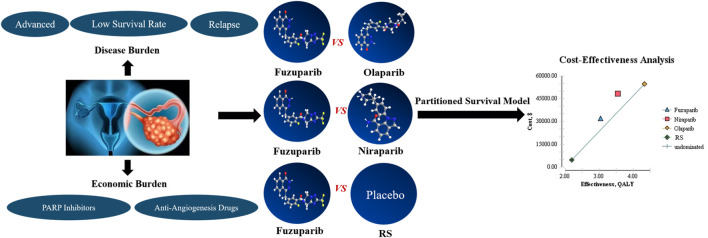

## 1 Introduction

Ovarian carcinoma (OC), including fallopian tube cancer and primary peritoneal cancer, is one of the most common gynecological cancers. The incidence of OC is rising yearly with the mortality ranking first in gynecologic tumors, which imperils the health of female (Smith, 2015). In 2016, data from National Cancer Center indicates that in China, 27,200 deaths and 57,200 new cases of OC were reported. The latter accounted for 20% of all new cases globally (Rongshou et al., 2022). The onset of OC is latent with 70% of patients diagnosed in advanced stage, and it is prone to recurrence. The main forms of treatment are surgery and postoperative platinum containing chemotherapy (Ledermann and Raja, 2011). For platinum sensitive relapsed patients, platinum containing combination chemotherapy schemes (carboplatin/paclitaxel, carboplatin/gemcitabine, carboplatin/docetaxel, cisplatin/gemcitabine, etc.,), with or without bevacizumab, are often used (Orr and Edwards, 2018). Nevertheless, platinum retreatment is accompanied with diminishing effectiveness and cumulative toxicity. Therefore, there is an urgent need for new treatment strategies that are well tolerated and able to effectively improve the progression-free survival (PFS) rate of patients suffering from relapsed OC.

Maintenance treatment retards the recurrence or reduces the incidence of relapse. For a long time, few drugs are applicable for maintenance treatment of recurrent OC, and the duration of bevacizumab maintenance treatment is limited. In recent years, poly (ADP-ribose) polymerase inhibitors (PARPis) have made breakthroughs as the maintenance treatment of recurrent OC with BRCA1/2 mutations, and have become a new treatment mode (Smith et al., 2015; Pujade-Lauraine et al., 2017). The advent of PARPis have changed the treatment modality of OC, making maintenance therapy an important part of the whole management process of OC, with milestone significance.

Loss-of-function mutations in BRCA1/2 have been found in 28.5% of patients among Chinese OC patients, which interferes normal repair of DNA double-strand breaks (Li et al., 2021a). PARPis enhance the efficacy of radiotherapy and chemotherapy with alkylating agents and platinum containing drugs *via* interrupting DNA single strand repairs and promoting tumor cells apoptosis through the mechanism that PARPis can block the alternative DNA repair pathway of BRCA1/2 mutated tumor cells resulting in synthetic lethality (Gong et al., 2020).

Currently, PARPis including olaparib (AstraZeneca), niraparib (Tesaro), fuzuloparib (Hengrui) have been approved by the National Medical Products Administration (NMPA) as maintenance treatment for platinum-sensitive, recurrent OC, which bring about delayed OC progression. Accordingly, the median PFS and quality of life of OC patients have also been notably improved (Wolford et al., 2020). In a double-blind, randomized, placebo-controlled, phase III trial (SOLO2/ENGOT-Ov21), olaparib maintenance therapy afforded in 19.1 months of median PFS *versus* 5.5 months in placebo [hazard ratio (HR), 0.30; 95% CI, 0.22–0.41; *p* < .0001] (Pujade-Lauraine et al., 2017), 51.7 months of median OS *versus* 38.8 months in placebo [hazard ratio (HR), 0.74; 95% CI, 0.54–1.00; *p* = .054[ (Poveda et al., 2021). In an international, multicenter, double blind, randomized, placebo-controlled, phase III trial (ENGOT-OV16/NOVA), the median PFS was significantly longer in the niraparib arm (21 months) than the placebo arm (5.5 months) in a niraparib cohort [hazard ratio (HR), 0.27; 95% CI, 0.17–0.41; *p* < .001] (Mirza et al., 2016). Also, in an ongoing, randomized, double-blind, placebo-controlled, phase III, multicenter study (FZOCUS-2) (Li. et al., 2022), fuzuloparib as a maintenance drug in patients with platinum-sensitive recurrent OC significantly prolonged PFS compared with placebo [12.9 vs. 5.5 months; hazard ratio (HR), 0.25; 95% CI, 0.17–0.36; *p* < .0001]. Thus, PARPis seem to be attractive options for the treatment of platinum-sensitive, recurrent OC. Nevertheless, considering the high treatment cost of PARPis, it is crucial to optimize the allocation of limited health resources. Several relevant studies have evaluated the cost-effectiveness (CE) of different PARPis in the treatment of OC(Guy et al., 2019; Armeni et al., 2020; Muston et al., 2020), but they are not assessed on fuzuloparib. This study aimed to investigate the CE of fuzuloparib compared with routine surveillance (RS), olaparib and niraparib for platinum-sensitive recurrent OC from the Chinese healthcare systems. The results provide appropriate standards and theoretical basis for national medical insurance policy and a reference for more cost-efficient clinical treatment options for patients.

## 2 Methods

### 2.1 Model structure

**FIGURE 1:**
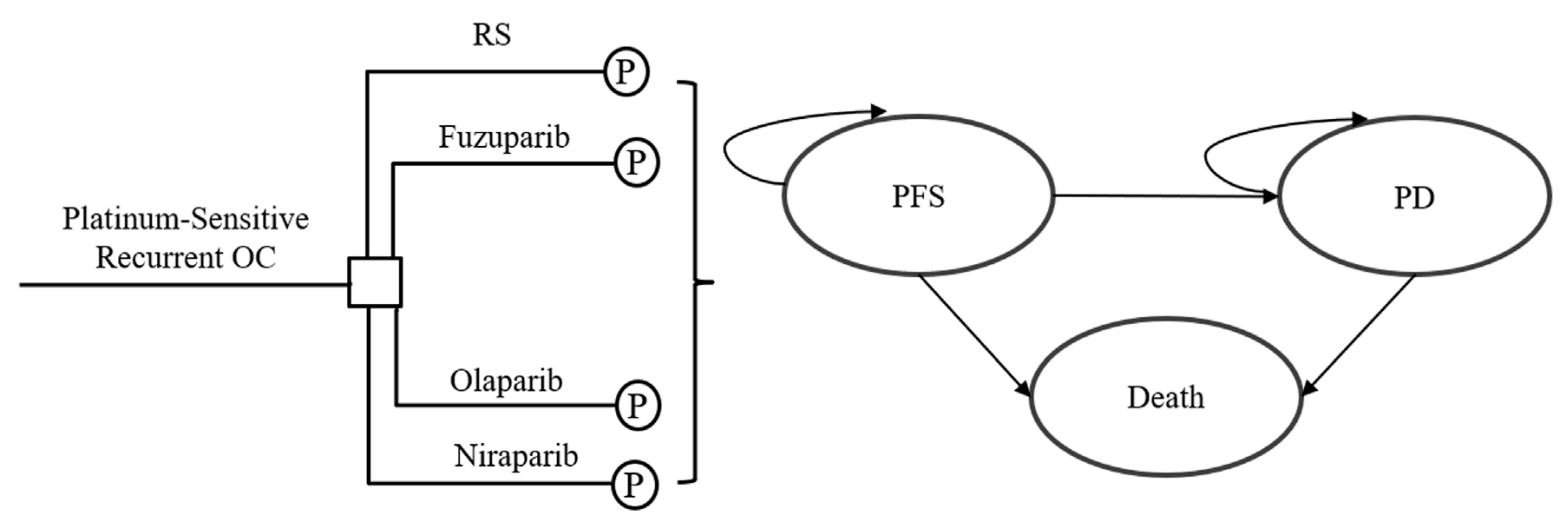
Model structure for platinum-sensitive recurrent ovarian carcinoma. OC, ovarian carcinoma; P, partitioned survival model; RS, routine surveillance; PFS, progression-free survival; PD, progressed disease.

The hypothetical target population for this analysis was patients with platinum-sensitive, recurrent OC, according to the patient characteristics of the clinical trials (Mirza et al., 2016; Pujade-Lauraine et al., 2017; Poveda et al., 2021; Li et al., 2022). A partitioned survival model with three health states was constructed to estimate the cost and treatment efficacy of therapy with RS, fuzuloparib, olaparib or niraparib (Wu and Shi, 2020). As shown in Figure 1, three mutually exclusive states were progression-free survival (PFS), progressed disease (PD), and death, respectively. It was assumed that all patients were in PFS state when entering the model. At the end of each cycle, the patient may stay in the original state or progress to the next state. FZOCUS-2 is a study completely based on the Chinese population, so the inclusion criteria and treatment regimen for the study target population were consistent with the FZOCUS-2 trial in this study. The study hypothesized that the patients entering the model had the same characteristics as the patients enrolled in the clinical trial, namely serous OC, primary peritoneal or fallopian tube cancer, or grade ≥2 endometrioid OC, platinum-sensitive after the last platinum dose of their penultimate line of chemotherapy, and an eastern cooperative oncology group (ECOG) performance status at a baseline of 0–1, and adequate organ function.

The GetData Graph Digitizer (version 2.20) was used to gather the data points from the PFS curves and OS curves, then data points were used to fit the following parametric survival function: Weibull, log-logistic, exponential, log-normal, gompertz and gamma. Akaike information criterion (AIC) and Bayesian information criterion (BIC) are both standards for measuring the goodness of model fitting, and the smaller value represents better goodness (Gao et al., 2021). In this model, the log-logistic and log-normal distribution are suitable for PFS and OS in RS group, respectively. Besides, The Weibull model was the most reasonable functions for extrapolating OS of niraparib group, and that the log-normal model was best for PFS and OS in other groups (see the Supplementary Appendix). Therefore, in our analysis, Weibull distribution, log-logistic distribution and log-normal distribution were used to calculate transition probability. Parametric survival curve was delineated by RStudio 2022.02.0 software. The distribution parameters of survival curve and the partitioned survival model were developed in TreeAge Pro 2011. General population mortality was derived from mortality tables of resident population in 2021 published by National Bureau of Statistics.

Recurrent OC is typically incurable with a low 5 year survival rate (Dottino et al., 2019; Ni et al., 2019). The time horizon was limited to 60 months, and each model cycle represents 28 days, which is in accordance to the treatment cycle of FZOCUS-2. The primary output of the model includes PFS quality-adjusted life years (PFS-QALYs) of the treatment scheme, and incremental CE ratios (ICERs). Based on the China guidelines for pharmacoeconomic evaluations (2020) (Guoen, 2020), 5% discount rate was adopted, and 1–3 times of Chinese *per capita* GDP in 2021 was used as the willingness-to-pay (WTP) threshold (12551.50$–37654.50$/QALY) (statistics, 2022). The incremental CE ratio was calculated to estimate the economic efficiency of treatment scheme.

### 2.2 Costs and utility values

The analysis was conducted from the perspective of the Chinese healthcare system, therefore, only direct medical expenses were considered, including the cost of drugs, follow-up test, best supportive care (BSC), terminal care and management of serious adverse effects (SAEs). The unit price of fuzuloparib, olaparib and niraparib in China was obtained from Shandong drug centralized procurement platforms, and other cost data was derived from literatures. All the costs were adjusted for inflation to reflect 2021 United States dollars according to Chinese Consumer Price Index (CPI) and based on the 2021 exchange rate (6.4515 RMB/United States dollar).

In order to simplify the model, only SAEs (≥3 grade according to NCI-CTCAE V5.0 criteria) with an incidence of more than 10% was considered in our research. It was assumed that adverse events occurred independently and probability remained constant over the 60-month time horizon. Discontinuation resulting from severe adverse reaction was not taken into account. For our base case, all adverse events of grade 3 and above were presumed to be incurred in the first cycle (Wu and Shi, 2020). All patients in the model were assumed to have regular laboratory testing with a carbohydrate antigen 125 (CA 125) and computed tomography (CT) every 2 months, and have a weekly complete blood count (CBC) for the first 4 weeks of treatment, followed by a CBC monthly, regardless of receipt of maintenance therapy strategy. The cost of BSC was the only cost included in this analysis after disease progression, and terminal care costs (TCC) were included in the final state. It is assumed that patients enter the model after receipt of platinum-based chemotherapy for their recurrence, and therefore clinical estimates and costs related to cytotoxic chemotherapy were not included. All information is listed in Table 1.

**TABLE 1:** Model parameters: baseline values, ranges, and distributions for sensitivity analysis.

Parameter	Expected value	Range	Distribution	Source
Drug costs				
Fuzuloparib/cycle	1394.73	1115.78∼1,394.73	fixed	Shandong drug centralized procurement platforms (2022)
Olaparib/cycle	1770.75	1416.60∼1770.75	fixed	Shandong drug centralized procurement platforms (2022)
Niraparib/cycle	1989.49	1,591.59∼1989.49	fixed	Shandong drug centralized procurement platforms (2022)
AEs costs				
Anemia	801.28	488.29∼1,114.27	gamma	Guy et al. (2019)
Thrombocytopenia	195.44	156.35∼234.52	gamma	Zhang et al. (2021)
Neutropenia	552.98	442.38∼663.57	gamma	Zhang et al. (2021)
Leukopenia	503.31	377.48∼629.14	gamma	Zhou et al. (2019)
Follow up monitoring cost				
CA 125 test	14.44	11.55∼17.33	gamma	Cheng et al. (2021)
Complete blood count	3.57	2.85∼4.28	gamma	Li et al. (2021b)
CT scan	95.53	76.42∼114.63	gamma	Li et al. (2021b)
BSC cost	126.49	101.18∼151.78	gamma	Zhou et al. (2019)
				Li et al. (2021b)
Terminal care cost	2,104.25	1,683.41∼2,525.10	gamma	(Zhou et al., 2019)
				Li et al. (2021b)
Utility				
PFS	0.84	0.67∼1.00	beta	Mirza et al. (2016)
PD	0.79	0.63∼0.95	beta	Mirza et al. (2016)
PFS life-years				
Fuzuloparib	1.08	0.93∼1.08	fixed	Li et al. (2022)
Olaparib	1.59	1.36∼2.14	fixed	Pujade-Lauraine et al. (2017)
Niraparib	1.75	1.46∼1.75	fixed	Mirza et al. (2016)
RS	0.46	0.32∼0.47	fixed	Li et al. (2022)
Discount	0.05	0.00∼0.08	fixed	Guoen, (2020)

AE, adverse event; CT, computed tomography; BSC, best supportive care; PFS, progression-free survival; PD, progressed disease; RS, routine surveillance.

In the clinical trials mentioned in the article, PFS was the primary endpoint. Consequently, in our model, PFS life-years were applied as the effectiveness measure for our base-case analysis and sensitivity analysis. QALYs were calculated by multiplying the PFS life-years with health-state utility values (HSUV), which was reported in the literature (Table 1). Regardless of the country assessed and the therapy applied, the utility values of the PFS and PS states in the same disease were the same (Li et al., 2021b).

### 2.3 Sensitivity analysis

One-way and probabilistic sensitivity analysis (PSA) were conducted using TreeAge Pro 2011 software to validate the model’s robustness when the results vary across a reasonable range. In one-way sensitivity analysis, the influence of parameter change on ICER value was calculated one by one according to the lower and upper limits obtained from credible intervals or a range of ±20% of the base case value (Wan et al., 2019), and the tornado graph was drawn using the obtained results. PSA was carried out *via* 1000 Monte Carlo simulations according to the distribution form of parameters (gamma distribution for cost, beta distribution for utility and incidence data) (Briggs et al., 2012). The ranges and distributions of the parameters used in the sensitivity analysis are given in Table 1.

## 3 Results

### 3.1 Base-case analysis

The costs for a 28-day medication were $1394.73, $1989.49 and $1770.75 for fuzuloparib, niraparib and olaparib, respectively, following the recommended dosing regimen in the package insert of drugs. In the base-case analysis, niraparib was associated with the longest PFS-life year (1.75), followed by olaparib (1.59), fuzuloparib (1.08) and RS (0.46). Compared with RS, the other three strategies showed positive effects in maintenance treatment of platinum-sensitive recurrent OC, and results of baseline CEA are show in Table 2. Total cost of fuzuloparib, niraparib and olaparib were $31628.10, $48183.48 and $54605.54. Compared with RS, the ICERs of olaparib, niraparib and fuzuloparib groups are all below WTP threshold, which is $23359.26, $31992.69 and $32216.08 respectively. Olaparib is superior to niraparib and fuzuloparib, with -$6,422.06 and -$16555.38 incremental costs and –0.79 and –0.51 incremental QALYs, respectively. Olaparib regimen is the highest cost therapy among all treatments, but with highest QALY, which means it is more cost-effective.

**TABLE 2:** Results of cost-effectiveness of fuzuloparib, olaparib and niraparib.

Treatment strategy	Total costs ($)	Total QALYs	Incremental costs ($)	Incremental QALYs	Incremental		ICER ($) *versus* baseline (QALYs)	ICER ($) incremental (QALYs)
					Costs ($)	QALYs		
RS	4,471.42	2.20						
Olaparib	54,605.54	4.34	50,134.12	2.15	50,134.12	2.15	23,359.26	23,359.26
Niraparib	48,183.48	3.55	−6,422.06	−0.79	43,712.06	1.36	32,216.08	Dominated
Fuzuloparib	31,628.10	3.05	−16555.38	−0.51	27,156.68	0.85	31,992.69	Dominated

QALY, quality-adjusted life years; ICER, incremental cost-effectiveness ratio; RS, routine surveillance.

### 3.2 Sensitivity analysis

**FIGURE 2:**
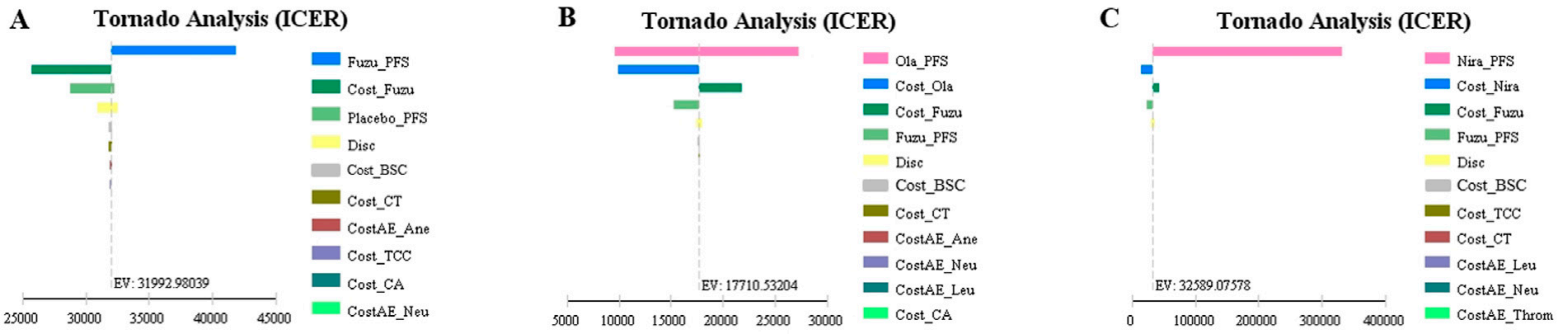
Tornado diagram of the one-way deterministic sensitivity analysis. **(A)** is the result of fuzuloparib maintenance therapy vs. RS, **(B)** is the result of fuzuloparib maintenance therapy vs. olaparib maintenance therapy, and **(C)** is the result of fuzuloparib maintenance therapy vs. niraparib maintenance therapy. PFS, progression-free survival; PD, progressed disease; AE, Adverse Event; CT, computed tomography; BSC, best supportive care; TCC, terminal care costs; RS, routine surveillance.

The tornado diagram showed that the ICERs of fuzuloparib vs. RS, fuzuloparib vs. olaparib, fuzuloparib vs. niraparib were all most sensitive to the PFS, followed by the cost of those medicines (Figure 2). In the fuzuloparib vs. RS, the ICER was higher than WTP threshold of $37654.50 per QALY when the PFS of fuzuloparib (0.93∼1.08) reduced to the lower threshold. In each group, other variables (such as discount rate, follow-up monitoring cost and adverse reaction treatment cost) had moderate or mild effects on ICER.

**FIGURE 3:**
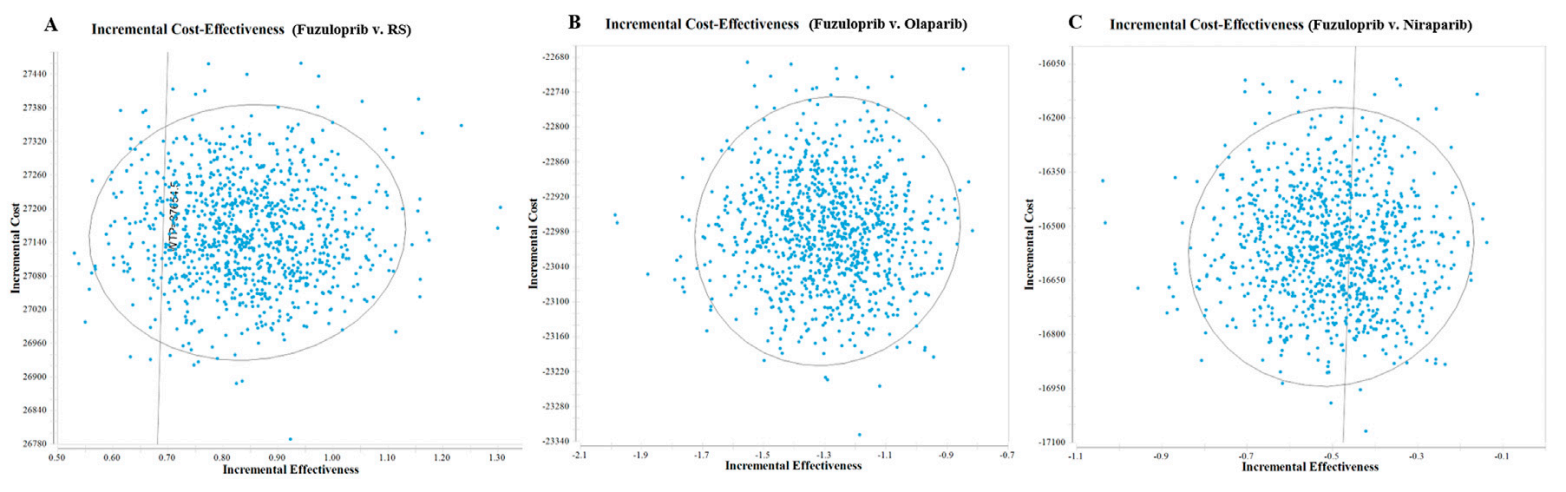
1,000 monte carlo simulation diagram of the probabilistic sensitivity analysis. **(A)** is the result of fuzuloparib vs. RS, **(B)** is the result of fuzuloparib vs. olaparib, and **(C)** is the result of fuzuloparib vs. niraparib. RS, routine surveillance.

The results of probabilistic sensitivity analysis suggested that the probability of fuzuloparib being CE compared with RS, oraparib and niraparib is 85.5%, 0% and 33.3% at a WTP threshold of $37654.50 per QALY, respectively (Figure 3). The results showed that olaparib was most cost-effective for patients.

## 4 Discussion

The clinical benefit from maintenance PARPis treatment according to several phase III trials has come to the foreground, but the high price is a barrier to its wide applications. Owing to the national policy, the price of PARPis have greatly reduced in China, even by more than 80%, which extremely reduces the threshold for the application of PARPis to cover more OC patients. Previous studies conducted by Wolford et al. (2020) and Leung et al. (2021) reported similar results for olaparib and niraparib, that olaparib was estimated to be more affordable and effective for patients with OC whether platinum-resistant or platinum-sensitive. While, Guy et al. (2019) and Zhong et al. (2018) reported that niraparib was more effective than olaparib in OC patients. Due to the particularity of the methodology itself, the results of pharmacoeconomic evaluation have poor transferability among different countries. To our best knowledge, this is an unprecedented study to evaluate the CE outcomes of fuzuloparib compared to RS, olaparib and niraparib for patients with germline BRCA1/2 mutation and platinum-sensitive recurrent OC from the perspective of the Chinese healthcare systems.

Based on our model, olaparib costs $31992.69, $17710.65 and $32589.34 per additional QALY gained compared with RS, olaparib and niraparib when only the health benefits in the PFS were taken into account. The PSA suggested a high probability up to 99.9% that fuzuloparib would be considered cost-effective at a WTP threshold of $50000 per QALY. Findings of the one-way sensitivity analysis showed that PFS of fuzuloparib was the most sensitive parameter compared to RS, this result indicated that patients gained more benefits with longer PFS. The cost of PARPis were also found to be a major driver of economic results. When the price of fuzuloparib decreased by 30%, the ICER for maintenance fuzuloparib decreased to a lower level than olaparib and niraparib in those people. On balance, the variables in the model are unlikely to affect the final outcome.

Currently, China implements drug pricing negotiation policy to help dynamically manage the national reimbursement drug list (NRDL) in order to alleviate the economic burden of patients and improve the affordability of drugs. Pharmacoeconomics provides an important reference in this process. And on the basis of the latest results of the drug pricing negotiations in 2021, fuzuloparib, olaparib and niraparib have successfully incorporated in NRDL, significantly increasing the availability and affordability of those drugs for patients. Drug treatment is still the main means in Chinese medical institutions at the moment. Diagnosis related groups (DRGs) payment is implemented in medical institutions and drug selection was paid more focus on the cost performance of drugs. Thereby, pharmacoeconomics is of great significance in hospital pharmacy.

Whereas, there are several limitations needing to be noted in our study. First, except for fuzuloparib, most of the people included were non-Chinese population or a small number of Chinese populations. Second, four PARPis have been approved in China at present, since the results of phase III clinical study of pamiparib has not been published, this study only included other three PARPis (fuzuloparib, olaparib and niraparib). Third, the utility values came from ENGOT-OV16/NOVA without Chinese population, instead of FZOCUS-2 including Chinese population (Mirza et al., 2016). Although health-related quality of life according to EQ-5D-5L were also assessed, the data have not been reported clearly in FZOCUS-2, so it is difficult to provide an accurate value, and the utility value reduced by adverse reactions was not taken into account. Fourth, the model adopts the simple model commonly used in the study of tumor pharmacoeconomics, without detailed classification of subgroups. Therefore, it seems to be difficult to accurately describe the disease progress. Finally, PFS was employed to measure QALY because the overall survival data on niraparib and fuzuloparib were not available during this study. Regardless of these limitations, however, the variables in the study didn’t affect the final outcome.

## 5 Conclusion

These estimates indicate that at a WTP threshold of $12551.50–37654.50/QALY, fuzuloparib was less cost-effective for patients with germline BRCA1/2 mutation and platinum-sensitive recurrent OC compared to olaparib, but was superior to niraparib from Chinese healthcare systems perspective. These findings may help clinicians to make optimal treatment decisions for those patients. Because of the great majority of data in this study from literatures, more high-quality clinical and Chinese economic real-world data are needed. Besides, mature OS data is also required to validate these results.

## Data Availability

The original contributions presented in the study are included in the article/Supplementary Material, further inquiries can be directed to the corresponding author.
