# Supplementary material for: Cost-effectiveness of fuzuloparib compared to routine surveillance, niraparib and olaparib for maintenance treatment of patients with germline BRCA1/2 mutation and platinum-sensitive recurrent ovarian carcinoma in China
Source: Front Pharmacol. 2023 Jan 4;13:987337. doi: 10.3389/fphar.2022.987337 (PMC9846494; doi:10.3389/fphar.2022.987337)
Supplement: Supplementary file 1 [file Table1.docx]

Cost-Effectiveness of Fuzuloparib Compared to Routine Surveillance, Niraparib and Olaparib for Maintenance Treatment of Patients with Germline BRCA1/2 Mutation and Platinum-Sensitive Recurrent Ovarian Carcinoma in China

Supplementary Table S1. The goodness of fit was based on AIC and BIC of KM curves

| Treatment | End Point | Goodness of fit | exponential | gamma | gompertz | weibull | loglogistic | lognormal |
| --- | --- | --- | --- | --- | --- | --- | --- | --- |
| Fuzuparib | PFS | AIC | 131.14 | 123.07 | 125.97 | 123.62 | 123.34 | 122.47 |
|  |  | BIC | 133.33 | 127.45 | 130.35 | 128.00 | 127.72 | 126.84 |
|  | OS | AIC | 155.66 | 149.75 | 150.81 | 149.82 | 149.87 | 149.57 |
|  |  | BIC | 158.38 | 155.20 | 156.27 | 155.27 | 155.33 | 155.03 |
| Niraparib | PFS | AIC | 495.68 | 492.73 | 497.43 | 494.07 | 490.30 | 486.53 |
|  |  | BIC | 498.61 | 498.58 | 503.28 | 499.93 | 496.15 | 492.38 |
|  | OS | AIC | 1396.70 | 1373.20 | 1380.07 | 1372.81 | 1375.46 | 1394.55 |
|  |  | BIC | 1400.83 | 1381.44 | 1388.32 | 1381.05 | 1383.70 | 1402.80 |
| Olaparib | PFS | AIC | 743.88 | 744.13 | 744.45 | 745.15 | 738.57 | 730.95 |
|  |  | BIC | 747.16 | 750.69 | 751.01 | 751.70 | 745.13 | 737.50 |
|  | OS | AIC | 1226.84 | 1200.15 | 1218.48 | 1204.88 | 1196.74 | 1191.43 |
|  |  | BIC | 1230.12 | 1206.71 | 1225.03 | 1211.44 | 1203.30 | 1197.98 |
| RS | PFS | AIC | 453.53 | 453.35 | 445.06 | 455.45 | 425.15 | 425.83 |
|  |  | BIC | 456.12 | 458.54 | 450.25 | 460.64 | 430.35 | 431.02 |
|  | OS | AIC | 672.81 | 657.89 | 670.05 | 661.41 | 654.32 | 651.47 |
|  |  | BIC | 675.40 | 663.08 | 675.24 | 666.60 | 659.51 | 656.66 |

AIC, Akaike information criterion; BIC, Bayesian information criterion; KM, Kaplan-Meier; OS, overall survival; PFS, progression-free survival.

Supplementary Table S2. The survival function formula and parameter value of optimal distribution of KM curve.

| KM curve | End Point | Optimal distribution | Survival function formula | value of the parameter | |
| --- | --- | --- | --- | --- | --- |
|  |  |  |  | μ | σ |
| Fuzuparib | PFS | log-normal | S(t)=1-φ[(log(t)-μ)/σ] | 2.735 | 0.736 |
|  | OS | log-normal | S(t)=1-φ[(log(t)-μ)/σ] | 3.877 | 0.896 |
| Olaparib | PFS | log-normal | S(t)=1-φ[(log(t)-μ)/σ] | 3.235 | 1.289 |
|  | OS | log-normal | S(t)=1-φ[(log(t)-μ)/σ] | 3.921 | 0.864 |
| Niraparib | PFS | log-normal | S(t)=1-φ[(log(t)-μ)/σ] | 2.827 | 1.138 |
| Placebo | OS | log-normal | S(t)=1-φ[(log(t)-μ)/σ] | 3.706 | 0.821 |
|  |  |  |  | λ | γ |
| Niraparib | OS | weibull | S(t)=exp(-λtγ) | 0.0439 | 1.254 |
| Placebo | PFS | log-logistic | S(t)=1/[1+(λt)^(1/γ)] | 5.862 | 1.830 |

KM, Kaplan-Meier; OS, overall survival; PFS, progression-free survival.
